# 24‐hour activity for children with cerebral palsy: a clinical practice guide

**DOI:** 10.1111/dmcn.14654

**Published:** 2020-08-27

**Authors:** Olaf Verschuren, Raquel Y Hulst, Jeanine Voorman, Sigrid Pillen, Nicole Luitwieler, Jeroen Dudink, Jan Willem Gorter

**Affiliations:** ^1^ UMC Utrecht Brain Center and Center of Excellence for Rehabilitation Medicine Utrecht University Utrecht the Netherlands; ^2^ Department of Rehabilitation Physical Therapy Science & Sports UMC Utrecht Brain Center University Medical Center Utrecht Utrecht the Netherlands; ^3^ Wilhelmina Children’s Hospital University Medical Center Utrecht Utrecht the Netherlands; ^4^ Sleep Medicine Center Kempenhaeghe, Heeze the Netherlands; ^5^ Department of Electrical Engineering Technical University Eindhoven Eindhoven the Netherlands; ^6^ OuderInzicht Parent Organization for Improvement of Parent Involvement in Research Amsterdam the Netherlands; ^7^ Rijndam Rehabilitation Rotterdam the Netherlands; ^8^ Department of Neonatology University Medical Center Utrecht Utrecht University Utrecht the Netherlands; ^9^ UMC Utrecht Brain Center University Medical Center Utrecht Utrecht University Utrecht the Netherlands; ^10^ CanChild Centre for Childhood Disability Research Department of Pediatrics McMaster University Hamilton Ontario Canada

## Abstract

The association between physical activity and health has been clearly established, and the promotion of physical activity should be viewed as a cost‐effective approach that is universally prescribed as a first‐line treatment for nearly every chronic disease. Health care providers involved in the care for individuals with cerebral palsy (CP) are encouraged to take an active role in promoting their health and well‐being. Balancing activity behaviours across the whole day, with improved physical activity, reduced sedentary time, and healthy sleep behaviours, can set up infants, preschool‐, and school‐aged children with CP for a healthy trajectory across their lifetime. However, most clinicians do not apply a systematic surveillance, assessment, and management approach to detect problems with physical activity or sleep in children with CP. Consequently, many children with CP miss out on an important first line of treatment. This article presents an evidence‐informed clinical practice guide with practical pointers to help practitioners in detecting 24‐hour activity problems as a critical step towards adoption of healthy lifestyle behaviours for children with CP that provide long‐term health benefits.

## Abstract 2

This article's abstract has been translated into Spanish and Portuguese.

Follow the links from the abstract to view the translations.

## Actividad durante las 24 horas para niños con parálisis cerebral: guía de práctica clínica

La asociación entre la actividad física y la salud se ha establecido claramente, y la promoción de la actividad física debe verse como un enfoque rentable que se prescribe universalmente como tratamiento de primera línea para casi todas las enfermedades crónicas. Se alienta a los proveedores de atención médica involucrados en la atención de personas con parálisis cerebral (PC) a que tomen un papel activo en la promoción de su salud y bienestar. Equilibrar las conductas de actividad durante todo el día, con una mejor actividad física, una reducción del tiempo sedentario y conductas de sueño saludables, puede preparar a los bebés, niños en edad preescolar y escolar con PC para una trayectoria saludable a lo largo de su vida. Sin embargo, la mayoría de los médicos no aplican un enfoque sistemático de vigilancia, evaluación y manejo para detectar problemas con la actividad física o el sueño en niños con PC. En consecuencia, muchos niños con PC pierden una importante primera línea de tratamiento. Este artículo presenta una guía de práctica clínica basada en evidencia con consejos prácticos para ayudar a los profesionales a detectar problemas de actividad durante las 24 horas como un paso crítico hacia la adopción de comportamientos de estilo de vida saludables para niños con PC que brindan beneficios para la salud a largo plazo.

## 24‐horas de atividades para crianças com paralisia cerebral: um guia para prática clínica

A associação entre atividade física e saúde tem sido claramente estabelecida,e a promoção de atividade física deve ser vista como abordagem custo‐efetiva que é universalmente prescrita como tratamento de primeira linha para toda doença crônica. Profissionais da saúde envolvidos no cuidado para indivíduos com paralisia cerebral (PC) são encorajados a assumir um papel ativo na promoção da sua saúde e bem estar. Balancear comportamentos ativos ao longo de todo o dia, com melhora da atividade física, redução do tempo sedentário, e hábitos de sono saudáveis, pode preparar lactentes, pré‐escolares e escolares com PC para uma trajetória saudável ao longo de sua vida. No entanto, a maior parte dos clínicos não aplica uma vigilância, avaliação e manejo sistemáticos para detectar problemas de atividade física ou sono em crianças com PC. Consequentemente, muitas crianças com PC deixam de receber uma importante primeira linha de tratamento. Este artigo apresenta um guia para prática clínica informado por evidências com pontos práticos para ajudar profissionais a detectar problemas de atividade física ao longo de 24 horas como um passo crítico em direção à adoção de comportamentos ativos de estilo de vida para crianças com PC que oferecem benefícios de longo prazo para a saúde.

**What this paper adds**

- The 24‐hour activity checklist detects problems in children with cerebral palsy (CP).
- A CP‐specific infographic facilitates effective 24‐hour activity counselling and education.

During childhood the body’s physical and cognitive development are rapid, and the attitudes and habits formed at an early age can set the stage for the child’s health in later life. Therefore, promoting an active lifestyle early on is key for optimal development and includes engaging in physical activity as well as minimizing sedentary behaviour. Owing to several factors, children with cerebral palsy (CP) usually have low levels of physical activity and spend prolonged times sitting.^1^ Given the high prevalence of inactive lifestyles and the concomitant risk of chronic health conditions in adults with CP, it is no surprise that recently published guidelines for children with CP are informed by the health implications of physical activity and sedentary behaviour.^1^

However, these recommendations might not be enough to optimize health and development. For typically developing children, there has been a recent shift towards healthy activity throughout the whole day. Referred to as the 24‐hour movement guidelines, the recommendations acknowledge that the entire activity continuum should be considered, which, in addition to physical activity and sedentary behaviour, also includes sleep.^2^ A child’s 24‐hour day can be spent in several activity domains. This means that a day could be divided into time spent sleeping, being sedentary, or being physically active at different intensities, while there is never a time when a child is not engaging in one of these activities. In other words, if time spent in one activity is increased, there is less time available in the day for the remaining activity domains. For example, 1 hour of sleep may be lost to compensate for an extra hour of sedentary behaviour, or, alternatively, for an extra hour of physical activity. In both examples the hour of sleep has been exchanged for another activity domain by exactly the same duration, but the two scenarios are likely to be quite different in their health implications. What may sound like a small shift in behaviour could have a significant long‐term impact on a child’s health trajectory; hence, the importance in considering the whole‐day activity pattern for children.

Emerging literature demonstrates a positive association between meeting the recommended hours of sleep and positive (brain) health outcomes in typically developing children, including global cognition.^3^ Healthy sleep seems especially important for more vulnerable children, yet the prevalence of sleep problems in children with CP is high.^4^ Following ‘the whole day matters’ philosophy, we need to adopt a proactive approach to ensure adequate hours of uninterrupted sleep in children with CP. The relation between physical activity and sleep is probably bidirectional where both acute and regular physical activity can improve sleep,^5^, ^6^ and sleep duration and quality may likewise influence physical activity behaviour.^7^ Getting adequate sleep helps typically developing children to feel energized in the morning and participate fully in their school day and after‐school activities. It allows them to be creative, play sports, socialize with friends, and be active during the day—which in turn helps children to sleep better. Exercise‐based interventions promote sleep efficiency and duration in the general population regardless of the mode and intensity of the activity,^8^ yet the effects of such interventions in children with CP may be less predictable. Although sleep‐time recommendations will probably be beneficial for all children with CP, they seem especially promising for those who are classified in Gross Motor Function Classification System (GMFCS) levels IV and V, who have limited capabilities and opportunities to be active in the moderate to vigorous activity spectrum of the movement continuum. Optimizing their sleep quality and quantity might be an effective intervention for lifetime health promotion, in particular when combined with the physical activity recommendations.

In summary, a balanced interplay between optimal yet feasible proportions of activity behaviours throughout the day (i.e. limited sitting, increased physical activity, and enough sleep) is recommended for a healthy life for all children; hence, the importance of adopting the 24‐hour activity guideline approach for children with CP. Key to promoting physical activity in the clinical setting is the use of tools in which every child’s 24‐hour habits are routinely assessed and recorded in their medical record. Those not meeting the recommendations, and their families, should be supported to optimize their 24‐hour activity levels and sleep pattern.

## THE ROLE OF PAEDIATRIC (RE)HABILITATION

The time is now to open a window of opportunity for 24‐hour activity assessment to be integrated into routine health care practice for children with CP. Paediatric physiatrists, (developmental) paediatricians, and other clinicians involved in the care of children with CP are particularly well‐positioned to address physical inactivity and sleep problems, because they often have an established relationship and routine with children with CP and their parents or primary caregivers. By incorporating physical activity across the 24‐hour activity continuum as a recognized vital sign (similar to height and weight), it can be documented and tracked routinely over time, facilitating meaningful and personalized counselling initiatives.

### Surveillance of sleep and physical activity problems

Although inquiring about sleep is widely advised in paediatric health care,^9^ sleep problems are still under‐reported and under‐recognized in children with CP.^10^ A recent qualitative study among parents of children with CP found that health care professionals rarely ask them about sleep issues during clinical encounters, and parents expressed a strong wish for sleep to receive more attention in paediatric rehabilitation settings.^11^ In addition, it is known that the physical inactivity and sedentary behaviour levels of children with CP are high and that these may require further attention to preserve and enhance their health and well‐being/development.^1^

Therefore, implementation of an assessment tool within a routine follow‐up or monitoring clinic is a good first option for clinicians interested in 24‐hour activity. We advocate a brief checklist with useful questions for discussing physical activity and sleep in children with CP. This checklist needs to be practical and feasible to implement in clinical practice. For a recent innovation project, we have developed a 24‐hour activity checklist, specifically for children with CP (Table S1, online supporting information). This tool includes questions carefully designed to determine whether a more thorough evaluation for a particular problem is warranted. Parents can complete the checklist at home before their appointment with the health care provider.

## DEVELOPMENT OF THE 24‐HOUR ACTIVITY CHECKLIST

For surveillance, clinicians need to be able to ask the right questions to recognize the signs of paediatric physical activity and sleep problems. Questions must be more specific than quickly asking parents whether their child has trouble being physically active or sleeping. For example, more than half of parents who have a child with a sleep disorder will deny such problems when asked directly using a general question only,^12^ whereas others may be unaware of what is normal or problematic sleep.^9^

For the questions related to sleep, a detailed description of the steps required for the development of paediatric sleep questionnaires was outlined by Spruyt and Gozal.^13^ The initial steps are important in providing evidence of content validity, namely the extent to which the questionnaire measures the intended construct and is appropriate for its intended use. Our 24‐hour activity checklist assesses the occurrence of different types of physical activity and sleep problems that are not necessarily correlated (i.e. a formative model); therefore, analysis about structural validity and internal consistency is not relevant and subsequently not reported for this checklist.^14^

### Exploratory interviews

Before the development of the checklist, 18 parents of children with CP (GMFCS levels I–V, aged 2–15y) were interviewed to explore their current situation, concerns, and needs about the care for sleep^11^ and physical activity of their child, to gain an initial understanding of the areas within the 24‐hour activity spectrum that warrant attention from a parental perspective.

### Item generation and content review

For children with CP, Capio et al.^15^ have systematically reviewed the literature on physical activity assessment measures, including questionnaires. Unfortunately, the current physical activity measurement tools are not practical for implementation in routine care. Specifically, for children with CP, the feasibility of existing questionnaires is limited owing to their length and the time required for the parents to complete them, as well as for the clinician to evaluate them.^16^ Moreover, existing instruments have not included sedentary behaviour (as part of physical activity) nor considered sleep to be part of the (24‐hour) physical activity spectrum. Using questions from previously validated questionnaires developed for other paediatric populations, such as the Family Nutrition and Physical Activity screening tool,^17^ and physical activity recommendations for children with CP,^1^ we have applied a structured approach with interviews involving both health professionals and parents of children with CP to adapt items and construct the new checklist. Existing paediatric sleep questionnaires were identified through literature review and reviewed by our project steering group with various backgrounds (i.e. paediatric rehabilitation, biomedical sciences, [paediatric] physical therapy, experts in the field of exercise physiology and sleep medicine, neonatology, and parenting). The steering group consisted of three researchers (aged 30–54y), seven clinicians (aged 38–60y), and three parents (aged 34–48y) to identify a range of questions, which related to symptoms relevant to identifying sleep problems in children with CP. We searched for questionnaires that could be used for children with CP across all ability levels. The following questionnaires were identified: the BEARS sleep screening tool,^18^, ^19^ the Sleep Disturbance Scale for Children,^20^ the Paediatric Sleep Questionnaire,^21^ and the Children’s Sleep Habits Questionnaire.^22^ These questionnaires have all been previously used in a variety of children with CP, and some have been validated in typically developing paediatric populations against objective measures, such as polysomnography.^23^ The health care professionals and parents liked the brief, simple nature of the BEARS sleep screening tool. The questions in the sleep section of the checklist were therefore based on items of the BEARS and supplemented with questions that could be more relevant for children with CP, such as pain/discomfort during the night. After examining existing questionnaires within the project steering group, relevant items were selected along with a response format (based on the Sleep Disturbance Scale for Children) that would be appropriate for all questions. A ‘don’t know’ option was added to help avoid non‐response. The addition of a ‘don’t know’ option can introduce some challenges to the interpretation and scoring of responses. However, since our aim was to develop a checklist (and not an outcome instrument), this was not considered a problem.

### Focus group

A focus group with three parents of children with CP (in GMFCS level I [aged 3y], level III [aged 8y], and level V [aged 12y]) was organized to discuss the developed checklist and, if necessary, modify the phrasing of the instructions and questions. Parents indicated that the checklist should also acknowledge parental sleep, which ultimately resulted in a checklist with 10 questions related to the sleep of the child and three questions related to the sleep of parents (each with an open‐ended question with room for remarks, questions, or concerns that parents might have had). In addition, parents valued the importance of incorporating questions for the children who were able to understand items such as how they thought they slept, and whether they thought being physically active was fun; these child‐oriented items were therefore added to the pilot checklist.

## CLINICAL EXPERIENCES AND IMPLICATIONS

In our innovation project, we have observed some clinical implications of the 24‐hour activity checklist, its use by clinicians, and its contributions to the care of children and their families.

After 12 months of pilot testing in three health care settings with quarterly evaluation meetings, we received mostly positive written feedback from participating parents (*n*=79; a response rate of 80% in a consecutive sample of patients) about usability, content, and clarity of the checklist. Their children represented a cross‐section of those with CP regarding their GMFCS levels (I–V) and ages (0–12y). In addition, verbal feedback was collected from paediatric physiatrists and developmental paediatricians in various settings: a children’s hospital (*n*=1), a school for special education (*n*=2), and a rehabilitation centre (*n*=6) in the Netherlands, about the use and applicability of the checklist in the clinical setting. The feedback and experiences of parents and clinicians can be summarized as follows. (1) The checklist, which was filled out by parents at home before their visit to the health care setting, was clear and easy to answer for parents. However, for some children it was not possible to answer the child‐oriented questions (e.g. the children were too young or unable to understand/answer). The children who were capable of filling out the child‐specific questions were very enthusiastic that they were asked about these issues. (2) On average, it took between 5 and 10 minutes to fill out the checklist, which was considered acceptable by all parents and children. (3) The paediatric physiatrists indicated that the questions yielded sufficient information related to physical activity and sleep to make an informed decision. (4) Reviewing and interpreting the checklist took clinicians no more than 2 minutes. (5) The checklist led to increased awareness and discussions with parents about 24‐hour activity behaviours. The fact that the parents filled out the checklist at home before the consultation resulted in a more prepared doctor’s visit; parents thought about possible consequences of not having enough activity throughout the day and the relation with their complaints. This facilitated the conversation between parents and health care professionals. In some cases, the appointments took longer than before implementation of the checklist. However, this was not considered a problem because the issues that were important for parents were now addressed. This process greatly increased the likelihood of identifying problems related to 24‐hour activities among children with CP, with referrals to specialized sleep clinics. (6) The findings were encouraging and made us decide to continue to keep the checklist in its current form, with additional instructions related to the questions for the child. See Table S1 for the final checklist.

Given that counselling on sleep can be time‐consuming and clinicians often feel incompetent to address sleep problems properly once encountered, many paediatric clinicians may inadvertently overlook sleep concerns.^24^ Moreover, many parents do not routinely share information about their child’s sleep,^10^ urging the need for incorporating questions about sleep into routine health assessment for children of all ages. Health care professionals are encouraged to proactively ask patients (and their caregivers) questions about their 24‐hour activity levels, and to provide specific counselling to assist with accessibility strategies for physical activity as well as suggestions for activity/exercise prescription and better sleep. Routine surveillance once a year using the checklist developed specifically for children with CP is a first step in the 24‐hour activity approach for this group of children. We have noticed that counselling serves as an approach to increase awareness of the 24‐hour continuum. For counselling and management, it is important that the clinician first informs parents of children with CP who have problems, or who are at risk of them, about the importance of sleep and physical activity, and educates them about the consequences that can occur if sleep or physical activity problems are not addressed. Follow‐up assessment needs to be performed when appropriate.

At present the 24‐hour activity checklist does not come with a validated algorithm or clinical care pathway. That said, clinicians are encouraged to take a common‐sense approach to determining the likelihood of a child (or parents) having one or more problems with sleep or physical activity. In other words, every question that has been answered negatively could be interpreted as a ‘red flag’ requiring follow‐up. For example, an endorsement of snoring on most days of the week (often or always) is suggestive and might be an indication to look for signs of obstructive sleep apnoea. In this situation, one single question will lead to the suspicion of a serious problem that should result in additional assessment. In case more than one question has been answered negatively, the clinician’s confidence that the child has a sleep problem requiring further investigation will probably increase. In our innovation project we used the following rule of thumb: once the paediatric physiatrist determined that there was a probability of one sleep or physical activity problem, the child was then referred for a comprehensive sleep or physical activity evaluation, respectively.

It is important to realize that the evaluation of sleep or physical activity problems requires a more thorough history of the child’s 24‐hour routine with a focus on bedtime habits, night‐time behaviour, naps, and daytime physical activity behaviour. This information can be obtained through (some form of) daily logs. Because of their easy accessibility and usability, such diaries may prove suitable for clinicians who are interested in physical activity and sleep duration, sleep schedule, and sleep‐related behaviours and interactions. However, when the focus is on sleep quality or sleep architecture, more sophisticated measures such as 24‐hour actigraphy for sleep and physical activity^25^ serve as a more reliable choice for those interested in objective physical activity and sleep assessment for extended periods in the child’s natural environment.

To make 24‐hour activity recommendations simpler both for clinicians to use and for parents (and children) to understand, we created an infographic (see Fig. S1, online supporting information). This infographic was created by an expert committee, including researchers, experts in the field of physical activity and sleep medicine, physicians, and parents. To improve inclusivity of the infographic, an additional round of feedback from parents of children with more severe CP, namely those in GMFCS levels IV and V, was undertaken. The primary goal of this infographic is to facilitate effective 24‐hour activity counselling and education for parents and professionals in paediatric health care. A recently published study explored how parents of children and young people with disabilities perceive the Canadian 24‐hour Movement Guidelines for Children and Youth.^26^ The results of this study indicate that although mothers of children and young people with disabilities have positive perceptions of the concept of the guidelines, the guideline recommendations and the brand messaging strategy are not inclusive or compatible with the abilities, needs, and previous experiences of children and young people with disabilities.^26^ It is important to realize that these guidelines are not based on any evidence specific to these children and young people. The guidelines that we present in this infographic are based on CP‐specific physical activity recommendations,^1^ use the generally accepted sleep recommendations,^2^ and take into account the suggestions to increase inclusivity by Handler et al.^26^ We call on everyone with responsibility for the care of young children with CP to join us in educating and empowering parents and clinicians on the importance of the 24‐hour activity approach for this group of children. This infographic should be used at every available opportunity to help all children with CP achieve the recommendations, and develop well and healthily.

In a recent study^27^ it has been shown that transitioning from a seated to a standing position contributes to the accumulation of light activity and reduces sedentary behaviour. This activity might be a feasible option for children with CP who are classified in GMFCS levels IV and V. However, for most clinicians this type of counselling for patients who need to increase physical activity is not routine practice, even though breaking up sedentary behaviour is generally accepted as an intervention. For more activities that can be used to increase the physical activity levels of children with CP, we highlight the Ability Toolkit, developed by Handler et al.^28^ This Toolkit (available at https://cdpp.ca/resources‐and‐publications/ability‐toolkit) is a resource for children and young people with a disability, and is meant to supplement the 24‐hour movement guidelines for typically developing children, similar to the guidelines for children with CP. The Ability Toolkit provides information relevant to adapting the guidelines to the unique movement abilities of children or teenagers with any type of disability. Some information may be especially useful for parents and guardians of children and teenagers with CP.

Unfortunately, there are limited data available on therapeutic approaches related to sleep in children with CP.^29^ However, a good starting point would be to promote good sleep hygiene.^30^ Sleep hygiene is defined as a set of sleep‐related behaviours that expose children to cues and activities that prepare them for and promote appropriately timed and effective sleep.^31^ These sleep promotion practices are grouped into four categories:^30^ (1) environmental (e.g. bedroom temperature and blackout curtains); (2) scheduling (e.g. regular bed‐ and wake‐times); (3) sleep practices (e.g. a relaxing bedtime routine, limiting screen time before bed); and (4) physiological (e.g. regular exercise and light exposure during the day).

Since successful management of chronic sleep disruption may decrease family stress and improve child functioning and development, it is timely for health care professionals who work with children and young people with CP and other developmental disabilities to change practice by using the 24‐hour activity checklist as a springboard for meaningful conversations. Despite the emphasis in the literature on physical activity over the past 10 years, recommendations for physical activity are only beginning to be applied by health care practitioners.^32^ We hope this 24‐hour activity checklist will accelerate change of practice.

### Moving forward with the 24‐hour activity checklist

In the past, clinical practice guidelines have been viewed as static documents. However, the science that informs clinical decision making continues to evolve. In this case, where the science related to sleep and physical activity in children with CP is rapidly evolving, guideline creation should best be viewed as a continuous improvement process with new studies reviewed and graded as they become available. It is important to realize that the checklist is based on the currently available literature and developed in co‐creation with parents and researchers. Although the checklist has been well received it does need further validation. Nevertheless, we hope that health care professionals will start using and incorporate the 24‐hour activity checklist into routine health assessment, experiment with and learn from its use, and provide us with feedback for continued improvement.

## CONCLUSION

In the care for children with CP there is little attention to healthy development through a 24‐hour approach. So, despite the emerging literature on the importance of a 24‐hour activity approach, there is a knowledge‐to‐action gap in this area for children with CP and their families. Tools such as the proposed 24‐hour activity checklist, have the potential to contribute to increasing awareness and changing practice.

Having an easy‐to‐use tool to identify problems within the 24‐hour activity continuum is only one step in the process of identifying and resolving paediatric sleep and physical activity problems in children with CP and their families. Once the results indicate a high probability of a problem, clinicians need to be prepared for follow‐up with counselling and a management plan. Often a multi‐ or interdisciplinary approach is essential, including medical specialists (sleep expertise, exercise medicine, child neurology, pain specialists) as well as service providers (including physical, occupational, and recreational therapists) and psychosocial expertise (developmental behavioural therapists, child life, early childhood educators, psychologists, social workers). It is hoped that a systematic integration of 24‐hour physical activity and sleep assessment into clinical settings can effectively identify a large population of at‐risk patients and act as a facilitator for comprehensive management as part of a broader‐based strategy to increase adoption of healthy lifestyle behaviours for children with CP.

## Supporting information


**Figure S1:** The 24‐hour activity guidelines infographic.

**Table S1:** The 24‐hour activity checklist
