# Supplementary material for: 24‐hour activity for children with cerebral palsy: a clinical practice guide
Source: Dev Med Child Neurol. 2020 Aug 27;63(1):54–9. doi: 10.1111/dmcn.14654 (PMC7754464; doi:10.1111/dmcn.14654)
Supplement: Supplementary file 1 — Figure S1: The 24‐hour activity guidelines infographic. [file DMCN-63-54-s001.pdf]

THE WHOLE DAY MATTERS

24-HOUR ACTIVITY GUIDELINES FOR CHILDREN AND ADOLESCENTS WITH CEREBRAL PALSY.

A GOOD NIGHT'S SLEEP, REGULAR EXERCISE, AND LIMITED SITTING ARE RECOMMENDED FOR PHYSICAL HEALTH, DEVELOPMENT, AND WELL-BEING.

SLEEP

HAVE ENOUGH HOURS OF GOOD QUALITY SLEEP, INCLUDING NAPS, WITH REGULAR BEDTIME ROUTINES.

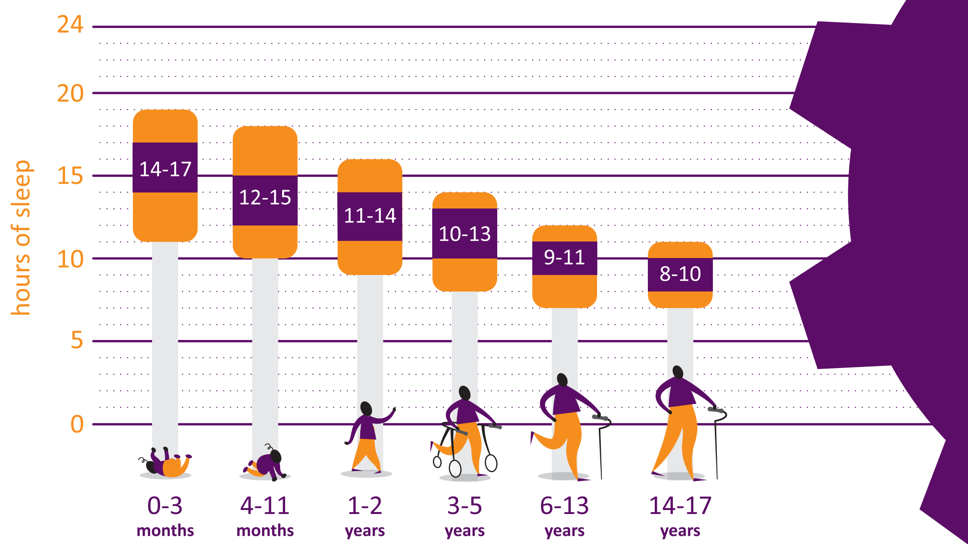

MOVE

EVEN LIGHT PHYSICAL ACTIVITY IS BETTER THAN NO PHYSICAL ACTIVITY AT ALL.

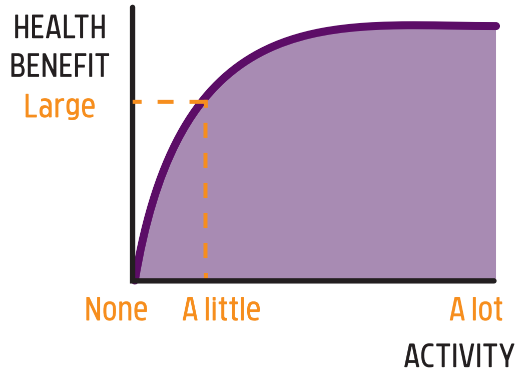

MEDIUM INTENSITY 60M per DAY AND HEAVY INTENSITY 20M 2-3 x per WEEK

MEDIUM TO HEAVY INTENSE PHYSICAL ACTIVITIES REQUIRE PHYSICAL EFFORT AND MAKES A CHILD FEEL MORE TIRED AND BREATHE HARDER THAN USUAL.

SIT

STRIVE FOR NO MORE THAN 2 HOURS PER DAY OF RECREATIONAL SCREEN TIME. LIMIT SITTING FOR EXTENDED TIME PER DAY.

CHANGE SEATED OR RECLINED POSITION (FREQUENTLY). IF APPLICABLE TO YOUR CHILD, TRY A STANDER.

NOTE THAT THERE ARE MANY SEATED ACTIVITIES THAT COUNT AS MEDIUM TO HEAVY PHYSICAL ACTIVITIES (E.G. WHEELCHAIR BASKETBALL, BOCCIA).

De Hoogstraat Revalidatie
UMC Utrecht
aandacht voor fysieke gezondheid bij kinderen met cerebrale parese
Center of Excellence for Rehabilitation Medicine Utrecht

IF YOU NEED SPECIFIC ADVICE, PLEASE CONTACT YOUR HEALTHCARE PROFESSIONAL.

DESIGN & ILLUSTRATION: HJALMAR HAAGSMAN 2020

OLAF VERSCHUREN 2020
O.W.VERSCHUREN@UMCUTRECHT.NL
