# Supplementary material for: 24‐hour activity for children with cerebral palsy: a clinical practice guide
Source: Dev Med Child Neurol. 2020 Aug 27;63(1):54–9. doi: 10.1111/dmcn.14654 (PMC7754464; doi:10.1111/dmcn.14654)
Supplement: Supplementary file 2 — Table S1: The 24‐hour activity checklist [file DMCN-63-54-s002.pdf]

Table S1: The 24-hour activity checklist

Instructions: Please answer the questions (reflecting on the past month) at home prior to your appointment with the healthcare professional. It approximately takes 10 minutes to complete the checklist. There are three questions that can be answered by your child if he/she is capable and willing to answer the questions (with your help), but this is not mandatory.

never=this never happens; seldom=this rarely happens, less than once a week; sometimes=less than half of the week; often=more than half of the week; always=almost every day or night

Sleep satisfaction	never	seldom	sometimes	often	always	don't know
1. Are you satisfied with the sleep of your child?						
**Initiating or maintaining sleep**	**never**	**seldom**	**sometimes**	**often**	**always**	**don't know**
2. Does it take more than 30 minutes before your child falls asleep?						
3. Does your child wake up more than 3 times a night, OR is your child awake for more than 20 minutes during the night?						
4. Do you think your child wakes up too early?						
**Snoring and pain/discomfort in bed**	**never**	**seldom**	**sometimes**	**often**	**always**	**don't know**
5. How often does your child snore at night?						
6. Do you think your child experiences pain or discomfort in bed?						
**Nightmares**	**never**	**seldom**	**sometimes**	**often**	**always**	**don't know**
7. How often does your child experience nightmares?						
**Fatigue**	**never**	**seldom**	**sometimes**	**often**	**always**	**don't know**
8. Does your child seem overtired or sleepy during the day?						
**Sleep medication**	**no**	**yes**				
9. Does your child use sleep medication/tablets (e.g. melatonin)?		My child uses:<br>…………………………………………………………………… (*name medication*)<br>(*dosage*) ………………… mg (number) ……….. times a week				
**Question related to sleep of your child**						
10. Do you have questions, remarks or concerns related to the *sleep of your child*?						
**Questions related to your own sleep**	**never**	**seldom**	**sometimes**	**often**	**always**	**don't know**
1. Are you satisfied with your own sleep?						
2. Do you think you have lack of sleep?						
3. Do you have questions, remarks or concerns related to *your own sleep*?						

**Physical activity: walking**	**yes**	**no**				
**1. Is your child able to walk (with or without an assistive device)?**						
**Physical activity: movement**	**<30 minutes a day**		**30-60 minutes a day**		**>60 minutes per day**	
**2. How many minutes does your child do something physically active when he/she has free time?**<br>*You can think of one of the following activities: Active play, walking, playing outdoors, running, cycling, swimming, dancing, horse-riding, playing sport (e.g. boccia, wheelchair basketball), (toddlers)gymnastics, playing on the floor, crawling, propelling a wheelchair.*						
**Fun in physical activity**	**yes**	**no**	**don't know**			
**3. Does your child like to be physically active? Does he/she experience fun in being active?**						
**Stimulating physical activity**	**yes**		**sometimes, but not always**		**no**	
**4. Do you know how you can help your child to be physically active?**<br>*Examples you can think about:*<br>*- <u>playing together</u>; playing or horsing around on the floor together, playing at the playground together, play sports together, walk the dog, do groceries etc.*<br>*- <u>moving independently</u>; crawling, walking (with or without a assistive device), riding a wheelchair, cycling, being mobile using a walker/handbike etc.*<br>*- <u>physical challenges</u>; For the children that are **able to walk** you can think about: walking stairs independently, walking long(er) distances, playing outdoors, etc.*<br>*For the children that are **not able to walk** you can think about: getting in and out of the wheelchair, activities on the floor/couch, sitting unsupported (under supervision) on the couch, playing on the floor, etc.*			Would you like some help/advice in this area?<br>☐ Yes, please<br>☐ No, thank you			
**Screen time (sedentary behaviour)**	**<1 hour a day**		**1-2 hours a day**		**>2 hours a day**	
**5. How many minutes a day does your child have "screen time" in his/her free time? (e.g. TV, computer, game system, or any mobile device with visual screens)**						
**Pain/fatigue**	**never**	**seldom**	**sometimes**	**often**	**always**	**don't know**
**6. Do you think your child experiences pain or fatigue while being physically active?**						
**Question related to the physical activity of your child**						
**7. Do you have questions, remarks or concerns related to the *physical activity of your child?***						

When possible, ask your child to answer the following questions (together with your help):

**Sleep**	
**1. How do you sleep at night?**	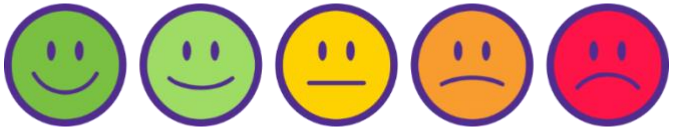<br>☐ ☐ ☐ ☐ ☐
**Physical activity**	
**2. Do you like to move?**	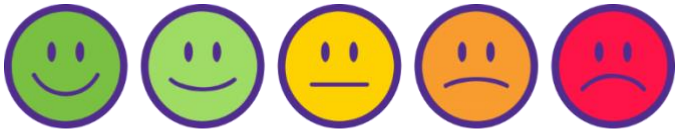<br>☐ ☐ ☐ ☐ ☐
**Comments**	
**3. Any additional comment(s) about your own sleep / physical activity?**	
